# Recurrent white thrombi formation in hemodialysis tubing: a case report

**DOI:** 10.1186/1471-2369-16-3

**Published:** 2015-01-15

**Authors:** Kiran P Sathe, Wee-Song Yeo, Isaac Desheng Liu, Sudha Ekambaram, Mohammed Azar, Hui-Kim Yap, Kar-Hui Ng

**Affiliations:** Shaw-NKF-NUH Children’s Kidney Centre, Khoo Teck Puat-National University Children’s Medical Institute, National University Health System, Level 12 NUHS Tower Block, 1E Kent Ridge Road, Singapore, 119228 Singapore; Department of Paediatrics, Yong Loo Lin School of Medicine, National University of Singapore, Singapore, Singapore

**Keywords:** Platelet activation, Renal dialysis, Thrombosis, Blood platelets, Hemodialysis, Home

## Abstract

**Background:**

While the appearance of red clots in the dialyzer is a common phenomenon in every hemodialysis unit, the occurrence of white thrombi in the tubing is relatively rare.

**Case presentation:**

We describe an adolescent male with recurrent white thrombi formation in the hemodialysis tubing. This patient had chronic renal failure from focal segmental glomerulosclerosis, but was no longer nephrotic at the time of the thrombi formation. He had a history of recurrent thrombosis of his vascular access. However, no pro-thrombotic risk factors could be identified. White particulate matter, measuring 1 to 3mm in size, and adherent to the arterial and venous blood tubing lines was found during the rinse back of a hemodialysis session. This was associated with a 60% decrease in his platelet count. Light microscopic examination of the deposits revealed the presence of platelet aggregates. He subsequently developed thrombosis of his arteriovenous graft six hours later. The white thrombi recurred at the next dialysis session, as well as six months later. These episodes occurred regardless of the type of dialysis machine or tubing, and appeared to resolve with an increase in heparin dose.

**Conclusion:**

Recurrent white thrombi formation can occur in the hemodialysis tubing of a patient with no identifiable pro-thrombotic factors. The white thrombi may be a harbinger of arteriovenous graft thrombosis and may be prevented by an increase in heparin dose.

## Background

Surface interaction between artificial material and blood during hemodialysis can lead to activation of complement, platelets as well as the coagulation cascade. The dialyzer membrane is often thought to be the main culprit in initiating these reactions, while the blood tubing in the circuit is generally believed to be inert. Biocompatibility of artificial materials used in hemodialysis is determined by the type of material, sterilisation mode and geometry [1, 2]. Poor biocompatibility can lead to decreased platelet counts and abnormal platelet function during dialysis [3], but acute thrombotic events are rare.

Here, we describe a case of recurrent white (platelet) thrombi formation in the hemodialysis tubing of a patient with no identifiable pro-thrombotic risk factors.

## Case presentation

The patient is a 16-year old Indian male who presented at the age of two years with nephrotic syndrome. This did not respond to initial steroid treatment and renal biopsy eventually revealed collapsing focal segmental glomerulosclerosis. No mutations in podocin and nephrin genes were found. He responded to a combination treatment regime comprising of prednisolone, cyclosporine A and mycophenolate mofetil. After a course of rituximab at the age of 14 years, he achieved remission and was maintained without steroids or other immunosuppressive therapy. Unfortunately, he relapsed one year later and progressed quickly to end-stage renal failure when he was 16 years old.

He was commenced on thrice-weekly conventional hemodialysis, using Gambro® AK 95™, via a permanent catheter. Unfractionated heparin was used for anti-coagulation. Platelet count remained normal during this period. His residual renal function declined rapidly, and three months after initiation of dialysis, he was no longer nephrotic. A brachio-axillary arteriovenous graft (AVG) was then created, but he developed four episodes of AVG thrombosis over a period of four months, necessitating repeated thrombectomies and angioplasties. Clopidogrel therapy did not prevent the recurrent thrombosis. Investigations revealed persistently elevated serum homocysteine level of 21.8 μmol/L (reference range 5 to 15 μmol/L) despite normal folate and vitamin B12 levels. Protein C, protein S, and antithrombin III levels were normal, and he was negative for factor V Leiden and antiphospholipid antibodies (anti-β2 glycoprotein, anti-cardiolipin antibodies and lupus anticoagulant). He had no other history of thrombotic events and the family history was similarly non-contributory. He had never smoked and was not obese (body mass index 20.4 kg/m^2^).

**Figure 1:**
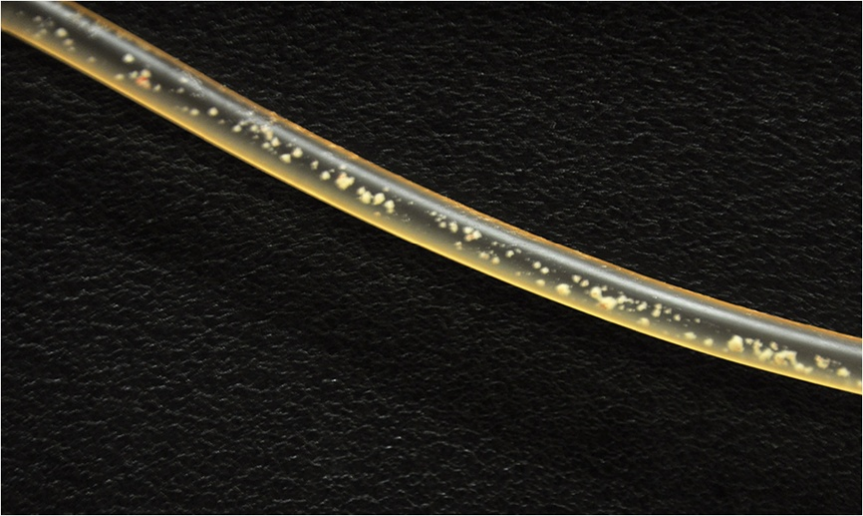
**White particulate matter measuring 1 to 3mm found adherent to the arterial and venous blood tubing lines after rinse back.**

The patient switched to home hemodialysis, using the NxStage® System One cycler, nine months after initiation of renal replacement therapy. His dialysis prescription was based on a target standard urea weekly Kt/V of 2.0 and involved five-hour sessions four times a week via his permanent catheter. A single bolus of unfractionated heparin 1,500 units was given at the beginning of each session. The first three sessions were uneventful. At the end of the fourth session, red clots were observed in the dialyzer. Accordingly, the bolus heparin dose was increased to 2,000 units at the following session. During rinse back at the end of the fifth session, white particulate matter measuring 1 to 3mm was found firmly adherent to the arterial and venous blood tubing (Figure 1). These were not present in the drip chamber or dialysate lines. Red clots were simultaneously noted in the dialyzer. The patient remained clinically well before, during and immediately after dialysis. The access, effluent and venous pressures during this session were not significantly higher than previous sessions. He presented to our nephrology service six hours later when he noticed decreased thrill in his AVG and was found to have AVG thrombosis, necessitating thrombectomy and stenting. Subsequent dialysis was carried out via conventional hemodialysis using Gambro® AK 95™. Unfractionated heparin was given at 1000 units and 500 units per hour as loading and maintenance doses respectively. During rinse back at the end of the next dialysis session, similar white particulate matter was again noted in the tubing, along with red clots in the dialyzer.

Several investigations were performed to elicit the cause of white particulate matter. Blood biochemistry and clotting function were all within acceptable limits. There was a 60% decrease in his platelet count (405 × 10^9^/L) two weeks prior; to 163 × 10^9^/L on the first day of white thrombi formation. At the second episode two days later, the platelet count increased slightly to 199 × 10^9^/L. His hemoglobin level (11.2 g/dL) and leukocyte count (8.27 × 10^9^/L) remained stable from baseline. Complement levels were normal. Antibodies for heparin-associated immune thrombocytopenia (HIT) were not measured. Analysis of the dialysate composition was within acceptable limits, with absence of free chlorine, chloramine, heavy metals and endotoxin. Dialysate microbiological cultures were negative. The polyvinylchloride tubing with the white particulate matter was cut and light microscopic examination of the deposits revealed platelet aggregates.

After the second episode of white thrombi formation, he was switched back to NxStage® System One home hemodialysis. An additional midway bolus of heparin 1,000 units was given for a few sessions with no further recurrence of white thrombi. Unfortunately, NxStage® home hemodialysis had to be discontinued after two months as the patient had poor hand dexterity following peripheral nerve injuries sustained during the creation of his AVG. During the 2 months of waiting for neurologic recovery, he was placed on Gambro® AK 95™ conventional hemodialysis with standard heparin doses. When he was converted back to NxStage® home hemodialysis, he had a newly created arteriovenous fistula. In an attempt to prevent fistula thrombosis, the anticoagulation regimen was changed to intravenous enoxaparin on dialysis days, and subcutaneous enoxaparin on non-dialysis days. His peak anti-Xa levels were maintained at 0.45 to 0.59 IU/ml on dialysis days. A third episode of white thrombi formation occurred while on NxStage® and the dose of enoxaparin was increased with no further recurrence. None of the other nine patients in the hemodialysis unit experienced the same phenomenon throughout the entire period.

## Conclusions

We report the repeated occurrence of white thrombi formation in the hemodialysis tubing of an adolescent male who probably had a pro-thrombotic tendency of undetermined cause. While the appearance of red clots in the dialyzer is a common phenomenon in every hemodialysis unit, the occurrence of white thrombi in the dialysis tubing is relatively rare. These episodes in our patient occurred regardless of the type of dialysis machine or tubing, and appeared to resolve with an increase in heparin dose.

Reports on reactions to hemodialysis tubing lines are scarce. In contrast, reactions to dialyzer membranes have been widely described. Such reactions can cause transient but significant intra-dialytic thrombocytopenia [3–5], and appear to depend on the manufacturer, polyvinylpyrrolidone content and sterilization method of the membrane [3, 6, 7]. Postulated mechanisms include biochemical interactions between blood and dialyzer membrane leading to complement and platelet activation. Activated platelets can form platelet-neutrophil, platelet-erythrocyte and platelet-platelet aggregates during dialysis [3, 8]. Despite the formation of such aggregates, reactions to dialyzer membranes do not generally lead to increased risk of thrombosis.

In a previous report by Watnick et al., white thrombi developed in the hemodialysis tubing of 21 out of 34 adult patients in a dialysis unit over a 5-week period [9]. This phenomenon did not occur after switching to dialysis lines from different manufacturers [9]. The white thrombi, however, recurred immediately after reinstatement of the dialysis lines from the original manufacturer, suggesting that dialysis tubing irregularities sustained during the manufacturing process could be the precipitating cause of white thrombi. These tubing defects could entrap microbubbles and enhance thrombogenicity.

In contrast, our patient was the only one who developed recurrent white thrombi in our hemodialysis unit over the 7-month period, regardless of the type of hemodialysis system (NxStage® and Gambro® systems). This supports our conclusion that it was his pro-thrombotic state that predisposed him to white thrombi formation, rather than the properties of any particular dialysis tubing. Similarly, in the series described by Watnick et al., a small subset of patients had recurrent white matter deposits despite the use of different machines on different occasions [9], suggesting the presence of unidentified patient-specific factors such as platelet function abnormalities.

The white thrombi formation described in our patient was most likely related to platelet activation, in view of the significant decrease in platelet count and the presence of platelet aggregates on microscopic examination of the white thrombi. We postulate that there was activation of the coagulation cascade, resulting in generation of thrombin which directly caused platelet activation and aggregation, and ultimately AVG thrombosis. Chronic renal failure is associated with changes in platelet function, coagulation and fibrinolytic factors such as thrombin-antithrombin III complex, fibrinopeptide A, D-dimer, von Willebrand factor and beta-thromboglobulin, leading to increased procoagulatory activity [10–13]. In addition, there is release of thromboxane A2 and adenosine diphosphate into the circulation, as well as increased platelet degranulation and aggregation [10–12]. Uremic patients have also been shown to have abnormalities in the platelet glycoprotein GPIb (receptor for von Willebrand factor), which were not corrected by hemodialysis or peritoneal dialysis [13]. Moreover, sera from patients on hemodialysis increased the expression of tissue factor in endothelial cells or smooth muscle cells and enhanced the platelet deposition in extracellular matrix [14, 15].

The degree of hyperhomocysteinemia in our patient was expected because of his renal failure [16, 17]. Patients on regular hemodialysis have higher serum homocysteine levels than healthy controls [18], possibly due to decreased homocysteine catabolism by the diseased kidneys [19]. Hyperhomocysteinemia is viewed as a non-traditional marker for cardiovascular disease, as it is associated with atherosclerotic events [18, 20]. While it may be part of the prothrombotic milieu in chronic renal failure [16, 17], its exact role in the thrombogenic process is not clear. Possible mechanisms include endothelial cell injury which in turn stimulates the local coagulation system [16, 21], or activation of the coagulation cascade via increased plasma tissue factor expression [22].

Our patient developed AVG thrombosis between the first and second episodes of the white thrombi formation, which occurred at consecutive dialysis sessions. While there was no evidence that the AVG thrombosis was directly caused by the white thrombi, it is conceivable that the AVG thrombosis was similarly related to the platelet activation. The temporal association of his thrombocytopenia, white clot formation and arterial thrombosis was reminiscent of HIT. Although HIT also involves platelet activation (related to antibodies to heparin-platelet factor 4 (*PF4*) complex), it was unlikely in our patient since the white thrombi occurred nine months after continuous heparin exposure, way beyond the time period when HIT typically occurs (usually five to ten days after initiation of heparin therapy [23]). For this reason, HIT antibody was not tested in our patient.

An increase in heparin dose appeared to prevent the recurrence of white thrombi in our patient. This was expected since heparin inactivates thrombin and therefore inhibits thrombin-induced activation of platelets. Low molecular weight heparins are probably as effective as unfractionated heparin in preventing extracorporeal circuit thrombosis [24].

In conclusion, this case highlights recurrent white thrombi formation in a dialysis patient with probable pro-thrombotic predisposition of undetermined cause. Such an episode may be a harbinger of AVG thrombosis and may be prevented by an increase in heparin dose.

## Consent

Written informed consent for the publication of this case report and any accompanying images has been obtained from the patient and his father. A copy of the written consent is available for review by the Editor of this journal.

## Abbreviations

(AVG): Arterio-venous graft
(HIT): Heparin-associated immune thrombocytopenia
(PF4): Heparin-platelet factor 4.
